# Abiotrophy

**Published:** 1902-04-26

**Authors:** 


### Hospital Clinics and Medical Progress.

### ABIOTROPHY.

We may speak of life as normal when the activities of the various parts of the body are so harmoniously correlated one with another that the functions of all are performed without excess and without waste. On the other hand we may speak of death as normal when it comes on not from the failure of this or that organ crippling the action of the rest, but from a gradual lowering of vitality all round, so that even to the last it would seem as if, feebly as the functions of any one part are carried on, the result is sufficient for the lessened demands of the other equally feeble organs. Such death is rare; for it usually happens that in consequence of the special incidence of extrinsic influences on certain regions, one part fails far before the rest. Local and unbalanced failure may, however, occur not from the special or irregular incidence of external injurious influences, but from the fact that certain tissues or tracts of the body have, perhaps even from birth, been less endowed than the rest with that attribute which we speak of as vitality, and that these tissues or tracts wear out sooner than is compatible with that evenly distributed decay which occurs in normal death. This condition of locally enfeebled vitality, with its coincident tendency to early degeneration, may undoubtedly arise as a result of inflammatory or other injurious influences, but in many cases it is equally evidently inborn, and it is to such inborn defect that we must look for the explanation of certain hereditary and inevitable degenerations which we are perhaps sometimes too ready to regard as diseases demanding treatment. In a lecture recently delivered at the National Hospital, Sir William Gowers pointed out that we want a name to express this conception—to describe this degeneration or decay which takes place in consequence of defect of vital endurance—and he suggested the use of the word "abiotrophy," formed by inserting the root of βιος after the negative particle in "atrophy," while if a more general term be desired the word "abiotic" might be used to express that which depends on defective vitality. Illustrations of this condition of abiotrophy may be found, he said, in the failure of the life of the hair follicles which is the essential cause of early baldness, or in that lesser degree of loss of function in which they fail to produce the normal pigment and thus cause early greyness of hair. Then there are certain conditions of the muscles which present striking forms of true abiotrophy, such as the various forms of idiopathic muscular atrophy, including pseudo-hypertrophic paralysis, in which the muscular fibres, after full development, cease to maintain their nutrition, slowly waste, and ultimately perish. However great the concurrent hypertrophy of the connective tissue and fat-bearing cells by which the pseudo-hypertrophy is caused may be, the essential element in the disease is the failure of vital endurance in the muscular fibres resulting from a congenital tendency. Coming, then, to the nervous system, one may find illustrations of abiotrophy in certain infantile atrophies, in certain spastic paraplegias, and in certain cases in which several members of the same family become blind, usually between the ages of 15 and 25, from slow failure of the fibres of the optic nerves. The best known form of abiotrophy, as affecting the nervous system, is, however, to be found in Friedreich's disease, the so-called "hereditary ataxy," which, whatever its varieties, must be regarded as the result of a degeneration of certain neurons from defect of vital power. Later in life, while general life may seem full of vigour, the nutrition of certain neurons may fail, and thus we may find various examples of abiotrophy. Those which most frequently thus decay are the spinal motor neurons, and with his usual wealth of illustration Sir William Gowers gave many examples of these forms of abiosis; but the point of interest in his lectures lay not merely in these, but in the clearness with which he laid down the general principle that many of the conditions which are often spoken of and perhaps regarded as instance of disease, and therefore fields for treatment, are really instances of abiotrophy. The recognition of this may, as he truly said, save one from many errors and prevent many mistakes both in prognosis and in treatment, while it may also enable one to perceive why in certain cases treatment fails, and may even prevent the useless prolongation of attempts to gain what cannot be.

